# Body weight status of school adolescents in Terengganu, Malaysia: a population baseline study

**DOI:** 10.1186/s12889-016-3911-2

**Published:** 2017-01-05

**Authors:** Aryati Ahmad, Nurzaime Zulaily, Nor Saidah Abdul Manan, Mohd Razif Shahril, Sharifah Wajihah Wafa Syed Saadun Tarek Wafa, Rahmah Mohd Amin, Engku Fadzli Hasan Syed Abdullah, Amran Ahmed

**Affiliations:** 1 Institute for Community Development & Quality of Life, Universiti Sultan Zainal Abidin, Gong Badak Campus, 21300 Kuala Nerus, Terengganu Malaysia; 2 Faculty of Informatics & Computing, Universiti Sultan Zainal Abidin, Tembila Campus, 22200 Besut, Terengganu Malaysia; 3 Faculty of Health Sciences, Universiti Sultan Zainal Abidin, Gong Badak Campus, 21300 Kuala Nerus, Terengganu Malaysia; 4 Faculty of Medicine, Universiti Sultan Zainal Abidin, Medical Campus, 20400 Kuala Terengganu, Terengganu Malaysia; 5 Institute of Engineering Mathematics, Universiti Malaysia Perlis, Pauh Putra Campus, 02600 Arau, Perlis Malaysia

**Keywords:** Body weight status, School adolescents, Terengganu, Malaysia

## Abstract

**Background:**

Body weight is highly associated with overall health status. Being severely thin or obese may impose the risk of many health problems. Early detection of body mass index (BMI) status may help to reduce the associated comorbidities. Although many studies in the literature have investigated the BMI of school adolescents in Malaysia, the data on status of body weight among school adolescents in suburban states like Terengganu is limited. This study aimed to describe the body weight status of the whole population of school adolescents in all seven districts in Terengganu, Malaysia.

**Methods:**

Using a cross-sectional study design, body weight and height were measured, and BMI was calculated and classified using WHO BMI-for-age Z-score. Data was obtained using the National Fitness Standard (SEGAK) assessment, which was uploaded in a specific Health Monitoring System (HEMS).

**Results:**

From a total of 62,567 school adolescents, 50.7% were boys and 49.3% were girls. Girls had significantly higher BMI than boys in age groups of 13 to 15 and 16 to 17 years old. Among boys and girls, there were significant differences in mean BMI of school adolescents between rural and urban school locations in all age groups (*p* < 0.001). There were also significant differences in BMI between boys and girls in all districts in Terengganu, except Kemaman and Kuala Terengganu, for all age groups (*p* < 0.001). Overall, the prevalence of thinness, normal, overweight and obesity were 8.4, 64.6, 15.0 and 12.0%, respectively. There were significant differences between BMI categories and genders in total participants, and within rural and urban school locations (*p* < 0.05). In all districts except Marang and Dungun, significant difference was also found between BMI categories and genders.

**Conclusion:**

The prevalence of thinness, overweight and obesity in Terengganu were substantial. In this study, BMI category was associated with gender, age, school location and district. However, the actual effects of these factors on the prevalence of thinness and obesity among this population demand further investigation.

## Background

For more than thirty years, malnutrition has been one of the public health concerns among children and adolescents, particularly in the least developed and developing countries. Severe thinness and being underweight have been associated with several health problems related to nutrition deficiency, leading to high mortality among children and adolescents [1]. The increase of food prices and the lack of food security have partly contributed to the food inadequacy among these countries. In spite of undernutrition, the epidemic of overweight and obesity among adolescents has also steadily increased, not only in developed but also developing countries. Apart from the biological and genetic factors, unhealthy diet and physical inactivity are the main mediators in the aetiology of obesity [2]. Obesity produces significant adverse effects on health status. With the increasing rate of obesity leading to increase in chronic diseases and poor health-related quality-of-life, the burden of its treatment has markedly escalated [3–5]. There are nearly 1.2 billion adolescents around the world, aged 10 to 19 years, who are facing growth and development challenges, and also at risk of adulthood disease morbidities, mainly due to poor nutrition and health status [6].

Body weight status, presented as body mass index (BMI), is an established and reliable indicator of body fatness for determining health status. BMI has been widely used at national and international levels to indicate malnutrition, either thinness or obesity, among all populations, including adolescents. Previous data from the Malaysian National Health Morbidity Survey (NHMS) 2011 has revealed that the national prevalence of thinness and obesity among adolescents aged 10 to 17 years were 9.7 and 5.7%, respectively [7, 8]. In Terengganu, 6.7% of adolescents were classified as thin, whereas 4.1% were obese. In addition, the national study also indicated that the prevalence of thinness and obesity was higher among urban areas compared to rural areas. Following that, the latest NHMS data (2015), which were recently released, reported that the national prevalence of thinness has decreased to 7.8%, whereas the prevalence of obesity has escalated to 11.9% among adolescents aged less than 18 [9]. Terengganu, however, showed an increase in both the prevalence of thinness and obesity, to 7.8 and 10.6%, respectively. Corresponding to the earlier report [7], prevalence of obesity and thinness were also found to be higher in urban areas compared to rural areas. Whether this data represents the actual body weight status of total school adolescents (aged 10 to 17) in suburban states like Terengganu is uncertain. The impacts of biological factors such as gender and age, with the influence of demographic factors like school locations and districts, on body weight status among this population are still debatable.

The present baseline study, as part of the Health of Adolescents in Terengganu study (HATs), aimed to provide the most recent state-level total population data with comprehensive descriptive analyses of body weight status among school adolescents in Terengganu, Malaysia. This data was anticipated to serve the Ministry of Education as well as the Ministry of Health for further policy or guideline development in relation to the health of school adolescents.

## Methods

### Study design and sampling

The present cross-sectional baseline study was conducted from November 2014 to June 2015 throughout Terengganu, Malaysia. The whole population of school adolescents, aged 10 to 17 years, from all public primary and secondary schools, were involved in the study. Schools within districts were classified as rural and urban by the Terengganu State Education Department (JPNT). The seven districts in Terengganu State were Besut, Dungun, Hulu Terengganu, Kemaman, Kuala Terengganu, Marang and Setiu. Terengganu is located on the East Coast of the Malaysian peninsular.

### Participants

A total of 512 schools were involved in this study, with 366 primary schools (*n* = 35,460) and 146 secondary schools (*n* = 27,107). Data were collected from a total of 67,519 adolescents. Of these, 62,567 had complete data and were included in this study. Participants included 31,708 male, and 30,859 female, adolescents, aged 10 to 17 years and attending public primary and secondary schools from seven districts throughout Terengganu. Participants were grouped into three school age groups, 10 to 12 years, 13 to 15 years and 16 to 17 years. These age groups refer to the standard public school staging system applied in Malaysia, which is also related to the examination system. In addition, participants were also classified into groups based on school location and the district that they live in.

### Data collection

Data on height, weight, gender and age were obtained from the first school term of 2015 National Fitness Standard (SEGAK) assessment test. The SEGAK programme is a standard physical fitness test to assess physical fitness level in primary and secondary school adolescents. In 2005, Malaysian Ministry of Education (MOE) initiated the SEGAK programme, and it was fully implemented nationally in 2008. The SEGAK test is a mandatory test, which is carried out twice a year (i.e. in March and August) by physical/health education (PE) teachers in schools. There are five main components involved, including measurement of BMI, step up, push-ups, partial curl-ups and a sit and reach test. Data of each student that completed the SEGAK test throughout Terengganu state were uploaded according to school by PE teachers into a web portal named Health Monitoring and Surveillance System (HEMS). The data of this study is strictly confidential and belongs to the Malaysian Ministry of Education. However, a complete report is due to be produced.

### Anthropometry measurements

Using a standardised protocol, height and weight were measured by the trained PE teachers in each school based on the reference material provided [10], and uploaded into the specific developed database in the HEMS web portal. Body mass and stature were measured using calibrated analogue health scales to the nearest 0.1 kg and 0.1 cm respectively. Data on height, weight, gender, and age were used to compute the BMI-for-age Z-score using WHO AnthroPlus software [11]. The age of each participant was calculated to the precise day by subtracting the date of birth from the date of measurement, while the BMI were calculated by dividing body weight in kilograms (kg) by height in metres squared (m^2^). At the time of data collection, all participants were apparently healthy and all measurements were taken in light sports attire, without shoes, during mornings or early afternoons. BMI categories were defined using age- and sex-specific cut-off points relative to WHO 2007 classifications [12]. The interpretation of the cut-offs classifies overweight as having z-score >+1SD, obesity as having z-score >+2SD and thinness as having z-score <−2SD.

### Statistical Analyses

SEGAK data were not available from several schools due to inappropriate data entry by the PE teachers. The results were examined for extreme values where reported BMI were below -5SD and exceeded +5SD, these were the arbitrary cut points by NHMS [7]. Descriptive statistics were presented as means, with their standard deviation or percentage of prevalence. This was used to describe the characteristics of the participants in terms of mean weight, height, age and BMI. Independent sample *t*-test was used to test the difference in mean of BMI between genders and school locations (rural vs. urban). Pearson’s chi square test was used to determine association between categorical variables. Analysis of variance (ANOVA) was used to test the difference in mean BMI between age groups and districts. Data was analysed using SPSS-IBM (version 22.0) (IBM Corporation, New York, USA). A two-sided *p* value of less than 0.05 was considered as statistically significant.

## Results

Participants’ distributions in genders, age groups, school locations and districts are presented in Table 1. A total of 62,567 school adolescents were involved in the study (50.7% male and 49.2% female), representing 81.1% of the total population of school adolescents aged 10 to 17 years in Terengganu. In total, 53.8% of school adolescents were from an urban, and 46.2% were from a rural, school location. Kuala Terengganu, as the capital city of Terengganu, has the highest proportion of school adolescents among all seven districts (41.3%), followed by Kemaman (13.9%), Besut (12.0%), Dungun (11.0%), Marang (7.4%), Hulu Terengganu (7.3%) and Setiu (7.0%).

**Table 1:** Subjects distribution

Variables	10 – 12 years			13 – 15 years			16 – 17 years			Overall		
	Boys	Girls	All	Boys	Girls	All	Boys	Girls	All	Boys	Girls	All
School location												
Rural	8393 (51.0)	8056 (49.0)	16449	4102 (51.3)	3887 (48.7)	7989	2023 (45.1)	2466 (54.9)	4489	14518 (50.2)	14409 (49.8)	28927
Urban	9738 (51.2)	9273 (48.8)	19011	4516 (53.0)	4012 (47.0)	8528	2936 (48.1)	3165 (51.9)	6101	17190 (51.1)	16450 (48.9)	33640
District												
Besut	2124 (50.6)	2075 (49.4)	4199	1167 (51.5)	1099 (48.5)	2266	498 (48.3)	532 (51.7)	1030	3789 (50.6)	3706 (49.4)	7495
Dungun	1870 (50.5)	1832 (49.5)	3702	992 (50.7)	966 (49.3)	1958	629 (50.2)	624 (49.8)	1253	3491 (50.5)	3422 (49.5)	6913
Hulu Terengganu	1111 (52.4)	1009 (47.6)	2120	784 (54.9)	644 (45.1)	1428	532 (50.8)	516 (49.2)	1048	2427 (52.8)	2169 (47.2)	4596
Kemaman	2905 (50.8)	2808 (49.2)	5713	904 (49.1)	936 (50.9)	1840	497 (44.5)	621 (55.5)	1118	4306 (49.7)	4365 (50.3)	8671
Kuala Terengganu	7780 (51.0)	7466 (49.0)	15246	3444 (54.4)	2886 (45.6)	6330	1967 (45.8)	2326 (54.2)	4293	13191 (51.0)	12678 (49.0)	25869
Marang	1119 (52.6)	1007 (47.4)	2126	816 (52.2)	748 (47.8)	1564	410 (44.2)	517 (55.8)	927	2345 (50.8)	2272 (49.2)	4617
Setiu	1222 (51.9)	1132 (48.1)	2354	511 (45.2)	620 (54.8)	1131	426 (46.3)	495 (53.7)	921	2159 (49.0)	2247 (51.0)	4406
Total	18131 (51.1)	17329 (48.9)	35460	8618 (52.2)	7899 (47.8)	16517	4959 (46.8)	5631 (53.2)	10590	31708 (50.7)	30859 (49.3)	62567

Data are n (%)

On average, the mean BMIs of total participants were 19.2 ± 4.6 kg/m^2^, 19.1 ± 4.6 kg/m^2^ and 19.3 ± 4.6 kg/m^2^, for boys and girls, respectively (Table 2). There was a significant association between age groups and BMI categories (*p* < 0.001). Girls had significantly higher BMI than boys in age groups of 13 to 15 and 16 to 17 years old (*p* < 0.001). No significant difference was found in mean of BMI between boys and girls in the 10 to 12 years old age group. Overall, there was a significant difference in mean BMI between rural and urban school locations (*p* < 0.001). Post hoc analysis indicated that mean BMI of urban boys and girls were significantly higher than their rural counterparts, but this finding was only restricted to the age group of 10 to 12 years old. The other age groups (13 to 15 and 16 to 17) showed no difference between urban and rural locations in both boys and girls. Conversely, within rural and urban school locations, mean BMI was only significantly different between boys and girls of age 13 to 15 and 16 to 17 years (*p* < 0.001), but not in age group of 10 to 12 years old (Table 2). By district, adolescents in Marang and Dungun shared the highest mean BMI, 19.4 ± 4.7 kg/m^2^ and 19.4 ± 4.5 kg/m^2^, respectively, whilst Kemaman had the lowest mean of BMI (18.9 ± 4.6 kg/m^2^). There were significant differences in mean of BMI between the seven districts (*p* < 0.001). Significant differences were also found between boys and girls aged 13 to 15 in Dungun, Hulu Terengganu, Kuala Terengganu, Marang and Setiu, and not significant in other age groups (Table 2).

**Table 2:** Anthropometric measurements by gender and age groups

	10 – 12			13 – 15			16 – 17			All		
	Boys	Girls	All	Boys	Girls	All	Boys	Girls	All	Boys	Girls	All
Age										12.7 ± 2.2	12.8 ± 2.3	12.7 ± 2.3
Height	137.4 ± 8.7	138.8 ± 9.1	138.1 ± 8.9	155.9 ± 9.9	151.9 ± 6.6	154.0 ± 8.8	165.0 ± 7.2	154.9 ± 5.9	159.6 ± 8.2	146.7 ± 14.3	145.1 ± 10.8	145.9 ± 12.7
Weight	34.5 ± 10.9	35.2 ± 10.8	34.9 ± 10.8	49.3 ± 14.2	48.0 ± 11.9	48.7 ± 13.1	57.9 ± 13.6	51.8 ± 11.7	54.7 ± 13.0	42.2 ± 15.4	41.5 ± 13.4	41.9 ± 14.4
BMI	18.0 ± 4.3	18.0 ± 4.2	18.0 ± 4.2	20.1 ± 4.6^a^	20.7 ± 4.6	20.4 ± 4.6	21.2 ± 4.5^a^	21.5 ± 4.4	21.4 ± 4.5	19.1 ± 4.6^a^	19.3 ± 4.6	19.2 ± 4.6
School location												
Rural												
Height	137.2 ± 8.7	138.6 ± 9.1	137.9 ± 8.9	155.9 ± 9.9	151.9 ± 6.6	154.0 ± 8.7	165.7 ± 7.1	154.8 ± 5.9	159.7 ± 8.4	146.5 ± 14.3	145.0 ± 10.8	145.7 ± 12.7
Weight	34.2 ± 10.8	34.7 ± 10.6	34.5 ± 10.7	49.4 ± 14.0	48.1 ± 11.7	48.7 ± 13.0	58.5 ± 14.0	51.7 ± 11.9	54.8 ± 13.3	41.9 ± 15.4	41.2 ± 13.4	41.6 ± 14.5
BMI	17.9 ± 4.2^b^	17.8 ± 4.1^b^	17.9 ± 4.2	20.1 ± 4.6^a^	20.7 ± 4.6	20.4 ± 4.6	21.2 ± 4.6^a^	21.5 ± 4.5	21.4 ± 4.5	19.0 ± 4.6^ab^	19.2 ± 4.6^b^	19.1 ± 4.6
Urban												
Height	137.5 ± 8.7	139.0 ± 9.1	138.2 ± 8.9	155.9 ± 10.0	151.9 ± 6.6	154.0 ± 8.8	164.5 ± 7.3	155.1 ± 5.8	159.6 ± 8.1	146.9 ± 14.2	145.2 ± 10.7	146.1 ± 12.7
Weight	34.8 ± 11.0	35.6 ± 10.9	35.2 ± 10.9	49.2 ± 14.3	48.0 ± 12.0	48.6 ± 13.3	57.4 ± 13.4	51.9 ± 11.6	54.6 ± 12.8	42.5 ± 15.4	41.8 ± 13.3	42.1 ± 14.4
BMI	18.2 ± 4.3	18.2 ± 4.2	18.2 ± 4.3	20.1 ± 4.7^a^	20.7 ± 4.6	20.4 ± 4.7	21.2 ± 4.4^a^	21.5 ± 4.4	21.4 ± 4.4	19.2 ± 4.6^a^	19.4 ± 4.6	19.3 ± 4.6
District												
Besut												
Height	137.3 ± 8.8	138.7 ± 9.6	138.0 ± 9.2	155.7 ± 10.4	152.2 ± 6.9	154.0 ± 9.0	165.8 ± 6.6	154.6 ± 5.9	160.0 ± 8.4	146.7 ± 14.3	145.0 ± 11.0	145.8 ± 12.8
Weight	33.9 ± 10.4	34.8 ± 10.5	34.4 ± 10.5	49.7 ± 14.4	48.1 ± 12.0	48.9 ± 13.3	57.9 ± 13.3	51.3 ± 11.9	54.5 ± 13.0	41.9 ± 15.4	41.1 ± 13.3	41.5 ± 14.4
BMI	17.8 ± 4.0	17.9 ± 4.1	17.8 ± 4.0	20.3 ± 4.7	20.7 ± 4.7	20.5 ± 4.7	21.0 ± 4.2	21.4 ± 4.4	21.2 ± 4.3	19.0 ± 4.5^c^	19.2 ± 4.6	19.1 ± 4.5
Dungun												
Height	136.7 ± 8.0	138.5 ± 8.6	137.6 ± 8.4	154.4 ± 10.0	151.1 ± 6.5	152.8 ± 8.6	164.2 ± 6.9	154.6 ± 6.1	159.4 ± 8.1	146.7 ± 14.0	145.0 ± 10.4	145.8 ± 12.4
Weight	34.0 ± 10.0	35.3 ± 10.2	34.7 ± 10.1	48.7 ± 14.0	48.0 ± 11.3	48.3 ± 12.7	57.8 ± 12.9	52.2 ± 10.7	55.0 ± 12.2	42.5 ± 15.2	42.0 ± 12.9	42.2 ± 14.1
BMI	18.0 ± 4.0	18.2 ± 4.1	18.1 ± 4.1	20.2 ± 4.7^c^	21.0 ± 4.6	20.6 ± 4.7	21.4 ± 4.3	21.8 ± 4.1	21.6 ± 4.2	19.2 ± 4.5^c^	19.6 ± 4.5	19.4 ± 4.5
Hulu Terengganu												
Height	137.6 ± 8.4	139.6 ± 8.7	138.5 ± 8.6	156.7 ± 10.1	151.9 ± 5.8	154.5 ± 8.8	164.9 ± 7.2	154.7 ± 5.8	159.9 ± 8.3	149.7 ± 14.5	146.8 ± 10.0	148.4 ± 12.7
Weight	33.7 ± 10.8	34.7 ± 10.3	34.2 ± 10.6	49.2 ± 13.6	47.7 ± 11.5	48.5 ± 12.7	58.1 ± 13.5	51.4 ± 11.8	54.813.1	44.0 ± 16.0	42.5 ± 13.3	43.3 ± 14.8
BMI	17.5 ± 4.2^d^	17.6 ± 3.9^f^	17.5 ± 4.1	19.9 ± 4.4^c^	20.6 ± 4.3	20.2 ± 4.4	21.3 ± 4.5	21.4 ± 4.6	21.4 ± 4.5	19.1 ± 4.6^c^	19.4 ± 4.5	19.2 ± 4.6
Kemaman												
Height	138.0 ± 8.8	139.1 ± 8.9	138.5 ± 8.9	154.9 ± 10.3	152.2 ± 7.2	153.5 ± 9.0	166.2 ± 6.9	155.1 ± 6.7	160.1 ± 8.7	144.8 ± 13.6	144.2 ± 10.8	144.5 ± 12.3
Weight	34.8 ± 10.8	35.2 ± 10.9	35.0 ± 10.9	48.7 ± 14.9	47.7 ± 12.2	48.2 ± 13.6	58.8 ± 14.9	52.2 ± 11.8	55.2 ± 13.7	40.5 ± 15.1	40.3 ± 13.3	40.4 ± 14.2
BMI	18.0 ± 4.3	17.9 ± 4.2	18.0 ± 4.3	20.1 ± 4.8	20.5 ± 4.5	20.3 ± 4.6	21.2 ± 4.8	21.6 ± 4.3	21.4 ± 4.5	18.8 ± 4.6	19.0 ± 4.5	18.9 ± 4.6
Kuala Terengganu												
Height	137.5 ± 8.8	138.8 ± 9.2	138.1 ± 9.0	156.4 ± 9.7	151.8 ± 6.7	154.3 ± 8.7	164.7 ± 7.6	155.2 ± 5.7	159.6 ± 8.2	146.5 ± 14.2	144.8 ± 10.9	145.6 ± 12.7
Weight	35.1 ± 11.3	35.4 ± 11.0	35.3 ± 11.2	49.7 ± 14.3	47.9 ± 11.9	48.9 ± 13.3	57.7 ± 13.9	51.9 ± 11.9	54.5 ± 13.2	42.3 ± 15.4	41.3 ± 13.4	41.8 ± 14.5
BMI	18.3 ± 4.5^e^	18.1 ± 4.3^g^	18.2 ± 4.4	20.1 ± 4.7^c^	20.7 ± 4.6	20.4 ± 4.6	21.2 ± 4.6	21.5 ± 4.5	21.4 ± 4.6	19.2 ± 4.7	19.3 ± 4.6	19.3 ± 4.7
Marang												
Height	136.0 ± 8.6	137.8 ± 9.1	136.8 ± 8.9	156.1 ± 10.1	151.9 ± 6.4	154.1 ± 8.8	166.5 ± 7.2	154.8 ± 5.7	160.0 ± 8.6	148.3 ± 15.2	146.3 ± 10.8	147.3 ± 13.3
Weight	33.4 ± 10.7	34.4 ± 10.6	33.9 ± 10.7	48.6 ± 13.8	48.9 ± 12.3	48.7 ± 13.1	59.1 ± 13.4	51.7 ± 12.3	55.0 ± 13.3	43.2 ± 15.9	43.1 ± 14.0	43.2 ± 15.0
BMI	17.8 ± 4.0	17.8 ± 4.0	17.8 ± 4.0	19.8 ± 4.6^c^	21.1 ± 4.8	20.4 ± 4.7	21.3 ± 4.5	21.5 ± 4.7	21.4 ± 4.6	19.1 ± 4.5^c^	19.8 ± 4.8	19.4 ± 4.7
Setiu												
Height	137.6 ± 9.0	139.3 ± 9.2	138.4 ± 9.1	156.2 ± 9.2	152.6 ± 5.9	154.2 ± 7.8	163.6 ± 6.9	154.7 ± 5.1	158.8 ± 7.5	147.2 ± 14.1	146.3 ± 10.5	146.7 ± 12.4
Weight	34.1 ± 10.5	35.0 ± 10.6	34.6 ± 10.6	48.9 ± 12.9	48.4 ± 11.6	48.6 ± 12.2	56.3 ± 12.7	51.8 ± 10.6	53.9 ± 11.8	42.0 ± 14.8	42.4 ± 13.3	42.2 ± 14.1
BMI	17.7 ± 3.9	17.8 ± 3.9	17.8 ± 3.9	19.8 ± 4.1^c^	20.7 ± 4.4	20.3 ± 4.3	21.0 ± 4.1	21.6 ± 4.0	21.3 ± 4.1	18.9 ± 4.2^c^	19.4 ± 4.4	19.1 ± 4.3

Data are mean ± SD (One-way ANOVA with Tukey’s post hoc test for comparison between districts)

^a^Significant difference in mean of BMI between genders in age groups (Independent sample *t*-test)

^b^Significant difference in mean of BMI between school locations within genders in age groups (Independent sample *t*-test)

^c^Boys vs girls in age group (*p* < 0.001) (Independent sample *t*-test)

^d^Hulu Terengganu vs Kemaman in boys aged 10–12 (*p* < 0.05)

^e^Kuala Terengganu vs Besut, Dungun, Hulu Terengganu, Kemaman, Marang and Setiu in boys aged 10–12 (*p* < 0.001)

^f^Hulu Terengganu vs Dungun in girls aged 10–12 (*p* < 0.05)

^g^Hulu Terengganu vs Kuala Terengganu in girls aged 10–12 (*p* < 0.05)

**Fig 1:**
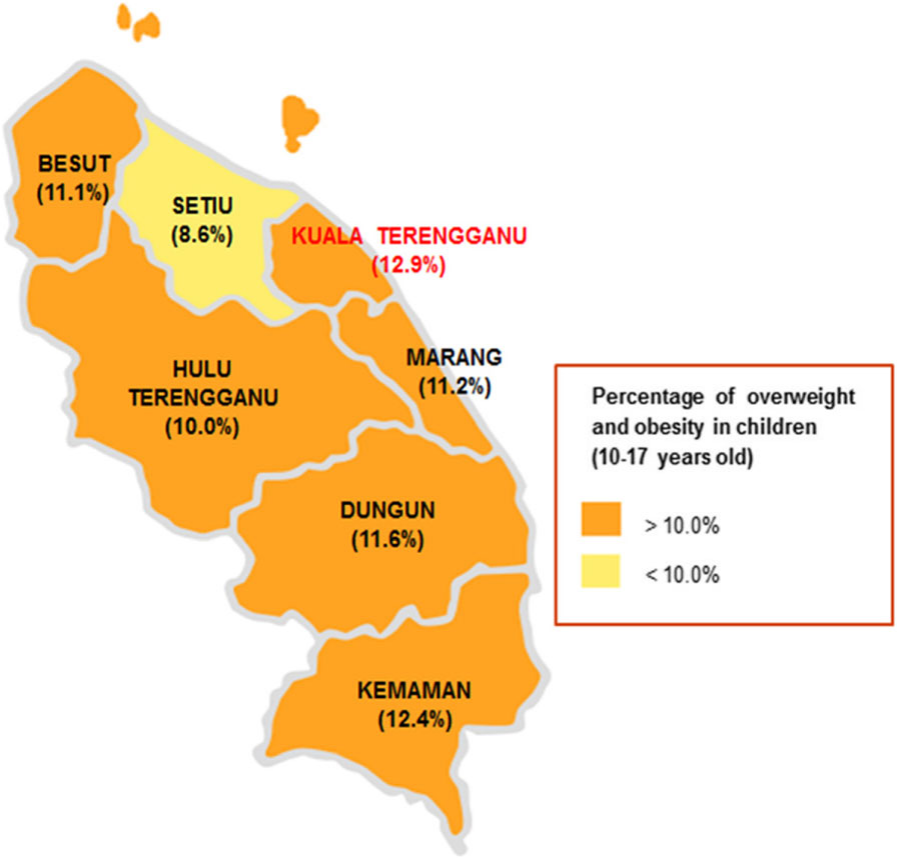
Map of obesity prevalence in Terengganu, Malaysia

In total, based on the WHO classification [12], the prevalence of thinness (<−2SD), normal (−2SD to +1SD), overweight (+1SD to +2SD) and obesity (>+2SD) were 8.4, 64.7, 15.0 and 12.0%, respectively (Table 3). While girls had higher prevalence of overweight, boys showed higher prevalence of thinness and obesity. This study showed a significant difference between BMI categories and age groups, mainly in the prevalence of thinness and obesity, although an inconsistent trend was seen in the prevalence of overweight (*p* < 0.001) (Fig. 1). Significant differences were also found between BMI categories and genders in all age groups (*p* < 0.001). Between school locations, the urban schools had a significantly higher prevalence of both overweight and obesity, with a lower prevalence of thinness compared to the rural schools. Significant differences were also found between BMI categories and genders in all age groups of urban and rural school locations. In addition, significant differences between BMI categories and school locations were found in boys (*p* = 0.001) and girls (*p* < 0.001) from age group of 10 to 12 years old. No difference was observed between rural and urban locations in other age groups.

**Table 3:** Percentage of BMI categories by gender and age groups

Variables	10 – 12 years				13 – 15 years				16 – 17 years				Overall			
	Boys	Girls	All	*p*-value^a^ (χ)	Boys	Girls	All	*p*-value^a^ (χ)	Boys	Girls	All	*p*-value^a^ (χ)	Boys	Girls	All	*p*-value^a^ (χ)
Thin	10.5	9.7	10.1	<0.001	7.4	5.6	6.6	<0.001	6.4	4.2	5.3	<0.001	9.0	7.7	8.4	<0.001
Normal	58.8	63.1	60.9	(216.3)	65.4	67.3	66.3	(51.4)	73.8	75.4	74.7	(45.4)	62.9	66.4	64.7	(289.6)
Overweight	14.6	16.1	15.3		15.0	17.1	16.0		11.2	13.5	12.4		14.2	15.9	15.0	
Obese	16.2	11.0	13.7		12.1	10.0	11.1		8.5	6.9	7.6		13.9	10.0	12.0	
School location																
Rural																
Thin	10.7	10.0	10.4	<0.001	7.2	5.6	6.4	<0.001	6.7	3.9	5.2	<0.001	9.2	7.8	8.5	<0.001
Normal	60.0	64.7	62.3	(110.0)	65.2	66.6	65.9	(18.8)	72.6	76.0	74.5	(26.8)	63.2	67.2	65.2	(140.1)
Overweight	14.0	15.3	14.6		15.4	17.4	16.4		11.8	13.3	12.6		14.1	15.5	14.8	
Obese	15.2	10.0	12.6		12.2	10.4	11.3		8.8	6.8	7.7		13.5	9.5	11.5	
Urban																
Thin	10.3	9.5	9.9	<0.001	7.7	5.6	6.7	<0.001	6.2	4.5	5.3	<0.001	8.9	7.6	8.3	<0.001
Normal	57.7	61.7	59.7	(107.8)	65.7	67.9	66.7	(33.5)	74.7	74.9	74.8	(22.5)	62.7	65.8	64.2	(150.6)
Overweight	15.0	16.8	15.9		14.7	16.9	15.7		10.8	13.7	12.3		14.2	16.2	15.2	
Obese	17.0	12.0	14.5		12.0	9.5	10.8		8.2	6.9	7.5		14.2	10.4	12.3	
*p*-value^b^	0.001	<0.001			0.639	0.527			0.441	0.667			0.28	0.013		
(χ)	(17.1)	(29.1)			(1.7)	(2.2)			(2.7)	(1.6)			(3.8)	(10.7)		
District																
Besut																
Thin	10.0	9.1	9.5	0.002	7.0	5.0	6.0	0.007	7.2	4.3	5.7	0.188	8.7	7.2	8.0	<0.001
Normal	61.5	64.5	63.0	(14.4)	64.3	69.5	66.8	(12.1)	74.1	76.3	75.2	(4.8)	64.0	67.7	65.8	(27.5)
Overweight	15.0	16.3	15.6		15.1	15.4	15.2		10.6	12.2	11.5		14.4	15.5	14.9	
Obese	13.5	10.0	11.8		13.6	10.1	11.9		8.0	7.1	7.6		12.8	9.6	11.2	
Dungun																
Thin	9.7	8.1	8.9	0.005	4.7	4.6	4.6	0.001	3.7	1.9	2.8	0.186	7.2	6.0	6.6	<0.001
Normal	59.4	63.3	61.3	(12.9)	69.6	67.0	68.3	(17.1)	76.6	76.6	76.6	(4.8)	65.4	66.7	66.1	(24.4)
Overweight	15.7	16.7	16.2		13.0	19.2	16.0		12.1	14.4	13.2		14.3	17.0	15.6	
Obese	15.2	11.9	13.6		12.7	9.3	11.0		7.6	7.1	7.3		13.1	10.3	11.7	
Hulu Terengganu																
Thin	13.1	10.7	11.9	<0.001	8.3	6.2	7.4	0.068	6.6	4.8	5.7	0.388	10.1	8.0	9.1	<0.001
Normal	62.3	66.7	64.4	(19.5)	65.1	68.0	66.4	(7.1)	75.6	76.9	76.2	(3.0)	66.1	69.5	67.7	(27.5)
Overweight	11.3	14.2	12.6		14.8	17.1	15.8		9.0	10.9	9.9		11.9	14.2	13.0	
Obese	13.4	8.4	11.0		11.9	8.7	10.4		8.8	7.4	8.1		11.9	8.3	10.2	
District																
Kemaman																
Thin	12.0	11.2	11.6	<0.001	8.4	6.7	7.6	0.061	8.5	4.7	6.4	<0.001	10.9	9.3	10.1	<0.001
Normal	57.5	61.7	59.6	(41.7)	63.3	66.6	64.9	(7.4)	71.8	72.9	72.5	(22.0)	60.4	64.4	62.4	(65.4)
Overweight	13.9	16.1	15.0		15.2	16.8	16.0		10.3	17.1	14.0		13.7	16.4	15.1	
Obese	16.6	11.0	13.8		13.2	9.9	11.5		9.5	5.3	7.2		15.0	10.0	12.5	
Kuala Terengganu																
Thin	10.4	10.2	10.3	<0.001	7.6	5.7	6.7	0.001	6.9	4.9	5.8	0.005	9.1	8.2	8.7	<0.001
Normal	56.5	61.4	58.9	(126.1)	65.0	67.2	66.0	(16.8)	72.6	75.2	74.0	(13.0)	61.2	65.3	63.2	(139.6)
Overweight	14.6	16.3	15.4		15.6	17.2	16.3		11.7	12.9	12.3		14.4	15.9	15.1	
Obese	18.5	12.1	15.4		11.8	10.0	11.0		8.7	7.0	7.8		15.3	10.7	13.0	
Marang																
Thin	9.9	7.8	8.9	0.004	8.7	5.1	7.0	0.03	7.3	3.3	5.1	0.025	9.0	5.9	7.5	<0.001
Normal	60.3	66.9	63.5	(13.6)	64.8	65.1	65.0	(8.9)	69.8	76.0	73.2	(9.3)	63.5	68.4	65.9	(23.2)
Overweight	16.0	15.5	15.8		15.1	17.2	16.1		12.9	12.4	12.6		15.1	15.4	15.2	
Obese	13.8	9.7	11.9		11.4	12.6	12.0		10.0	8.3	9.1		12.3	10.3	11.3	
Setiu																
Thin	7.9	8.0	7.9	0.16	7.4	6.5	6.9	0.742	4.0	3.4	3.7	0.098	7.0	6.5	6.8	0.066
Normal	65.6	68.3	66.9	(5.2)	67.9	67.3	67.6	(1.2)	79.1	74.9	76.9	(6.3)	68.8	69.5	69.2	(7.2)
Overweight	15.4	15.5	15.4		15.5	17.6	16.6		10.6	16.2	13.6		14.5	16.2	15.3	
Obese	11.0	8.3	9.7		9.2	8.7	8.9		6.3	5.5	5.9		9.7	7.8	8.7	
*p-*value^c^	<0.001	<0.001			0.05	0.346			0.036	0.016			<0.001	<0.001		
(χ)	(125.5)	(64.9)			(28.8)	(19.8)			(30.2)	(33.1)			(151.1)	(92.5)		

Data are %

^a^BMI categories versus genders in age groups (Pearson’s chi-square test)

^b^BMI categories versus school locations in boys and girls (Pearson’s chi-square test)

^c^BMI categories versus districts in boys and girls (Pearson’s chi-square test)

According to districts, the prevalence of overweight was highest in Dungun (15.6%), followed by Setiu (15.3%), Marang (15.2%), Kemaman and Kuala Terengganu (15.1%), Besut (14.9%) and Hulu Terengganu (13.0%). Nevertheless, the prevalence of obesity was found to be highest in Kuala Terengganu (13.0%), followed by Kemaman (12.5%), Dungun (11.7%), Marang (11.3%), Besut (11.2%), Hulu Terengganu (10.2%) and Setiu (8.7%). In contrast, Kemaman had the highest prevalence of thinness (10.1%), followed by Hulu Terengganu (9.1%), Kuala Terengganu (8.7%), Besut (8.0%), Marang (7.5%), Setiu (6.8%) and Dungun (6.6%). There were significant differences between BMI categories and genders in adolescents aged 10 to 12 years old from all districts (*p* < 0.05) except Setiu. In the age group of 13 to 15 years old, significant differences were found between BMI categories and genders in adolescents from Besut, Dungun, Kuala Terengganu and Marang (*p* < 0.05). Among adolescents aged 16 to 17 years old, significant differences between BMI categories and genders were only found in the districts of Kemaman (*p* < 0.001), Kuala Terengganu (*p* = 0.005) and Marang (*p* = 0.025). There were also significant differences between BMI categories and districts in boys and girls aged 10 to 12 (*p* < 0.001 and *p* < 0.001) and 16 to 7 years old (*p* = 0.036, and *p* = 0.016).

## Discussion

The findings of this study were analysed and compared with those of other international and national studies, as well as those of other states in Malaysia. For national and state comparison, NHMS 2011 was referred to because the latest NHMS 2015 data reported pooled findings among adolescents and children aged less 18 years [7, 9]. This study was aiming to report data on school adolescents aged 10 to 17 years. The results present the descriptive statistics on mean BMI and prevalence of thinness and obesity among adolescents in different school locations and districts in Terengganu. To our knowledge, this is the only study with an attempt to provide data on body weight status of whole population of school adolescents in all states of Malaysia. In total, the means of BMI increased as age increased, however, this was consistently plotted in normal range.

### Differences in BMI between boys and girls

Mean of BMI significantly differed between genders in secondary school-age adolescents. This can be explained by several factors, including physiological changes and difference in lifestyle between genders at this age [13]. Girls at this age may have significantly higher BMI compared to boys as a result of rapid growth and physical changes associated with sexual maturation and puberty. Girls generally experience the growth spurt associated with puberty two years earlier compared to boys [14]. In addition, girls tend to engage in less physical activity, such as sports, compared to boys in their secondary schools [15–17]. The significant differences between genders were primarily contributed by several districts, including Dungun, Hulu Terengganu, Kuala Terengganu, Marang and Setiu. The association between districts and the difference in mean BMI between genders may be explained by differences in level of urbanisation between districts and lifestyle between genders in each district [18]. In agreement with the SEANUTS study [19], no significant difference in mean BMI between boys and girls in the primary school-aged group was found. This may potentially be due to less difference in lifestyle, mainly with regards to total energy intake [20].

### Differences in BMI between school locations

In agreement with the SEANUTS and NHMS 2011 studies, mean BMI was significantly higher among boys and girls from the urban location compared to the rural location, particularly in the 10 to 12 year old age group [8, 21]. Rapid advance in environmental, social and physical developmental changes after the urbanisation process has led to the increase in sedentary lifestyle among adolescents [22, 23]. Due to higher socioeconomic status, these urbanised adolescents are more engaged with gadgets and technology, which has reduced their time spent involved in physical activity. In addition, the transition towards Westernised diets, mainly in processed and fast foods, which are high in calorie, sugar and fat content, has a significant role in the increased BMI among adolescents in the urban areas [24]. Nevertheless, significant differences in mean BMI were also observed between several districts. Boys from the district of Kuala Terengganu had significantly higher mean BMI compared to other districts. Kuala Terengganu acts as the capital city of Terengganu and is primarily made up of urban and developed areas, which may have contributed to the increased problem of unhealthy lifestyles.

### Prevalence of obesity and thinness in Terengganu, Malaysia and its association between gender, age group and school location

According to the z-score BMI categories, overall prevalence of obesity in this study (12%) was higher than the national prevalence and Terengganu for 2011 [8]. The prevalence of obesity in Terengganu has increased by two-fold in 2015. In comparison with other state like Kelantan, the prevalence of overweight and obesity was considerably higher, by 13.9% [25]. Other Asian developing countries, such as China and India, also reported an increasing trend of childhood obesity, from 6.4% in 1991 to 7.7% in 1997 and 4.9% in 2003 to 6.6% in 2005, respectively [26]. In spite of the increased prevalence of overweight and obesity among these adolescents, the prevalence of thinness in Terengganu has also increased from 6.7% in 2011 to 8.4% in 2015 [8]. It was lower compared to the national prevalence in 2011 (12.2%) but higher than the latest prevalence in 2015 (7.8%) [7, 9]. The findings from this study indicated a contradictory trend compared to the national study in the prevalence of thinness in association with age. The latest NHMS study showed an increasing trend of thinness with increasing age [8]. Conversely, in agreement with the same study, the trend of obesity prevalence reduced as age increased.

While there was a significant difference between BMI categories and genders in all age groups, the prevalence of adolescents with normal BMI increased with age. Parallel with the SEANUTS study [19], girls had lower prevalence of thinness and obesity but higher prevalence of overweight compared to boys. Contrary to the present findings, the NHMS 2011 study [7] has reported that the prevalence of obesity increased whilst thinness decreased as age increased in both genders. The reason for this difference was speculated to be due to the difference in sampling method. The NHMS study implied household method whereby this study targeted adolescents in schools. Nonetheless, the prevalence of overweight increased in the age group of 13 to 15 before it decreased to the lowest prevalence in 16 to 17 years old. A similar trend was found among underweight boys and girls in Kuala Lumpur [27]. Parallel with the NHMS (2011) data for Terengganu, the prevalence of both thinness and obesity were lower among girls compared to boys in all age groups [8]. This is also consistent with the findings from two previous national studies [28, 29]. In contrast, no difference was found between genders in the prevalence of overweight and obesity among adolescents in Turkey [30].

As observed in this study, the trend of obesity was slightly higher in the urban areas compared to the rural areas for boys and girls from 10 to 12 years old age group. Conventionally, most of the nutritional studies in Malaysia and other countries showed a higher prevalence of obesity in the urban areas. However, thinness was more prevalent in the rural areas. Interestingly, no difference in this prevalence between rural and urban areas was detected in this study, especially in the older age groups. While the urban adolescents might have higher mean BMI, the number of adolescents classified as obese may not differ from the rural areas. The current socioeconomic transition has shifted the lifestyle of adolescents in the rural areas to mimic their urban counterparts. The influence of television advertisements and social media, as well as easy access to food outlets, has led to the increased consumption of fast and processed foods among rural adolescents [24, 31]. Likewise, data from the NHANES study has indicated that more rural adolescents were obese compared to the urban adolescents [32]. On the other hand, the number of adolescents who indulge in inactive leisure time activities has increased in the rural areas [24, 33]. Traditionally, thinness was more associated with rural areas; however, findings from this study suggested no difference in thinness prevalence between rural and urban areas. Developing countries like Malaysia are now facing the epidemic of over-nutrition, rather than under-nutrition, which may have shifted public attention towards the underweight adolescents in both areas. The influence of different districts on the prevalence of both thinness and obesity was also uncertain except for the youngest group of adolescents. This might be due to comparable developmental issues between districts. There are limited data to compare between districts in any other state of Malaysia.

These findings are sufficient to indicate the steady increment of obesity prevalence among adolescents, at an alarming rate in comparison to previous years. Indirectly, it reflects the inadequacy of health awareness, attitude, and practice among the adolescents, their parents and related environments. Comorbidities of obesity, particularly the non-communicable diseases, will certainly have implications for the expensive cost of long-term medical treatments. While previous epidemiological studies have identified the major risk factors of childhood obesity, tackling each individual risk factor has to start promptly at early stages. In spite of the ‘westernised’ problem of obesity, undernourished adolescents do exist. Underweight adolescents are indeed vulnerable to many health consequences, such as nutritional deficiency, stunting, infection, prolonged recovery, and death. There is a gap between these two kinds of malnutrition among adolescents in the country. Reducing both rates is crucial to ensuring the successful development of the country. Many activities have to be well-planned, and all of them need to involve each contributing society or body, including parents, school, stakeholders and community, in order to ensure a healthy environment for the growth of adolescents.

#### Strength and limitation

This study presents the data of body weight status of all school adolescents age 10 to 17 years old in the whole of Terengganu. It represents the actual figure of both thinness and obesity prevalence in Terengganu, as compared to other available national and state-level studies, thus reducing the risk of under- or over-estimation of prevalence. However, this study compiled the body weight and height data that were measured by the PE teachers from each school, which may have introduced an inter-researcher variability. Even so, these teachers were fully trained and provided with calibrated and validated measurement tools, which may, thus, have reduced the potential bias introduced by the different teachers. The main reason to include teachers in the measurement process is because they are responsible for the continuity of this surveillance and monitoring process throughout the adolescents’ school years. This study was meant to serve as baseline for future larger scale surveillance and monitoring studies at the national level.

## Conclusions

In conclusion, prevalence of thinness and obesity were both considerably increased in comparison to previous data. The epidemic of obesity among adolescents has become an emerging and alarming issue. It has become the major global health issue in both developed and developing countries. It leads to increased financial burdens due to escalating cost of treatment, particularly during adulthood, for obesity-related non-communicable diseases, such as metabolic syndrome, diabetes mellitus, hypertension and non-alcoholic fatty liver disease. Lifestyle modification, primarily through healthy diet and physical activity together with effective health awareness and behaviour change, should be promptly implemented as early as possible before the problem of obesity and its comorbidities become untreatable. Although obesity is the current serious health issue, underweight adolescents should receive equal attention, in order to prevent later adverse health implications. Provision of healthy meals at school for the poor students is considered as one of the effective ways to tackle this problem. Overall, this baseline data is important to provide evidence of actual body weight status among whole school adolescents in Terengganu, Malaysia. It is also sufficient to alert the accountable stakeholders to initiate and develop relevant intervention programs to overcome related health problems. Nonetheless, this study should also be followed up by a longitudinal prospective study to investigate the relative risk owned by these adolescents.

## Abbreviations

ANOVA: Analysis of variance
BMI: Body mass index
HATs: Health of adolescents in terengganu study
HEMS: Health monitoring System
JPNT: Terengganu State Education Department
kg: Kilograms
m^2^: Metre squared
MOE: Ministry of Education
NHANES: National Health and Nutrition Examination Survey
NHMS: National Health Morbidity Survey
PE: Physical/health Education
SD: Standard deviation
SEANUTS: South East Asia Nutrition Survey
SEGAK: National fitness standard
SPSS: Statistical package for the social sciences
UHREC: Universiti Sultan Zainal Abidin Human Research Ethics Committee
WHO: World Health Organization
